# Lithotomy on a Man Seventy-eight Years of Age

**Published:** 1843-01

**Authors:** 


### LITHOTOMY ON A MAN SEVENTY-EIGHT YEARS OF AGE.

As an encouragement to the aged to submit to this operation, not less than an item of passing surgical news, we may mention that Professor Gross lately operated on a gentleman 78 years old. This is an uncommon age for the operation, though it has often been successfully performed at that time of life. The diagnosis, on sounding, was a small calculus, which proved to be the case; but the operator discovered, immediately after its extraction, that others remained, but were encysted. With some difficulty the cyst was broken open, when upwards of fifty were brought away—averaging a cherry in size. As usual, in such cases, they had facets, worn by their attrition against each other. This nest had evidently not been detected by the sound. No bad symptoms followed, and the patient is now restored. D.

